# Kayser–Fleischer ring with keratoconus: a coincidence? A case report

**DOI:** 10.1186/s12886-020-01463-4

**Published:** 2020-05-13

**Authors:** Peike Hu, Lin Lin, Zhiyi Wu, Xiuming Jin, Hailong Ni

**Affiliations:** The Second Affiliated hospital of Zhejiang University School of Medicine, Eye Center, Hangzhou, Zhejiang China

**Keywords:** Keratoconus, Wilson’s disease, Kayser-Fleischer ring

## Abstract

**Background:**

It is rare for hepatolenticular degeneration [Wilson’s disease (WD)] to occur along with keratoconus (KC). In our report, a teenager was diagnosed with WD because of the discovery of Kayser–Fleischer (KF) ring in the cornea, and concomitant KC was found.

**Case presentation:**

A 19-year-old male was diagnosed with KC due to a rapid decline in visual acuity within a short period of time. Ocular examination revealed the presence of ring-shaped, dense, brown sediment at the Descemet membrane of the bilateral limbus cornea, exhibiting characteristics similar to those of KF ring. Then, the patient was referred to the Department of Neurology and diagnosed with asymptomatic WD. During the next 5 years of follow-up, the patient has worn RGP lenses, routinely taken drugs that inhibit copper absorption and promote copper excretion, and maintained a low-copper diet. He has never exhibited obvious systemic symptoms associated with WD, such as neurological, mental, or hepatic dysfunction, and the color of the KF ring has grown obviously lighter. Moreover, the morphology of the cornea has stabilized.

**Conclusion:**

Only one report of WD combined with KC was found in the literature. So far, there is no evidence of a correlation between the occurrence of the two diseases. However, a low-copper diet and active copper-reducing therapy may have played a role in stabilizing the patient’s condition in this case.

## Background

Keratoconus(KC) is associated with some syndromes and diseases, such as Down syndrome, Leber congenital amaurosis, and certain connective tissue lesions [1–3]. It is rare for hepatolenticular degeneration [Wilson’s disease (WD)] to occur along with KC. KF ring is a characteristic ocular sign of WD, as it is reported in the majority of WD patients with neurological symptoms and approximately half of those patients without neurological symptoms. In our report, a male teenager was diagnosed with WD because of the discovery of KF ring in the cornea, and KC was found at the same time. This is the second reported case of WD combined with KC so far [4].

## Case presentation

**Fig. 1:**
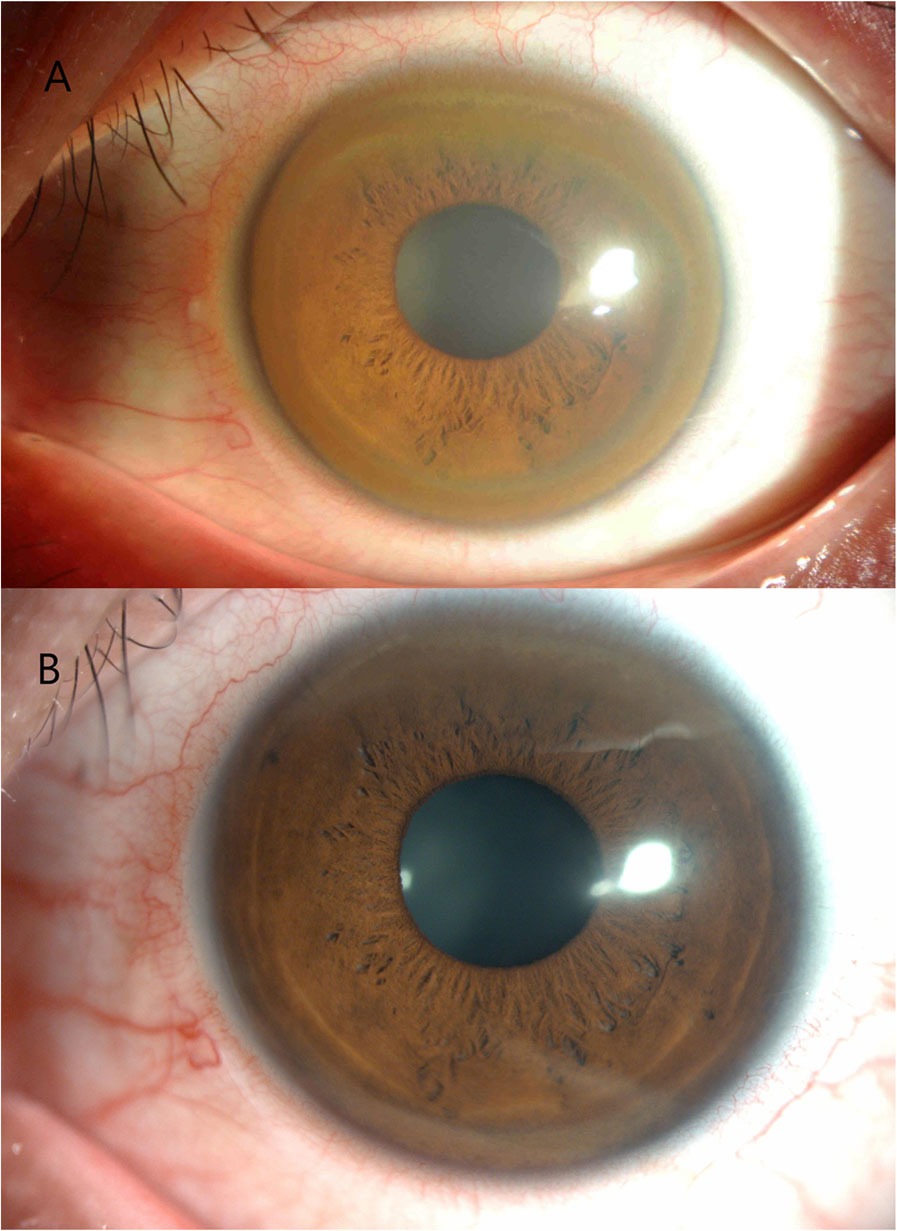
Part **a**: The KF ring was prominent, and the corresponding iris texture was almost invisible. Part **b**: The cornea at the most recent visit, where the KF ring had become markedly lighter and the iris texture was clearly visible

**Fig. 2:**
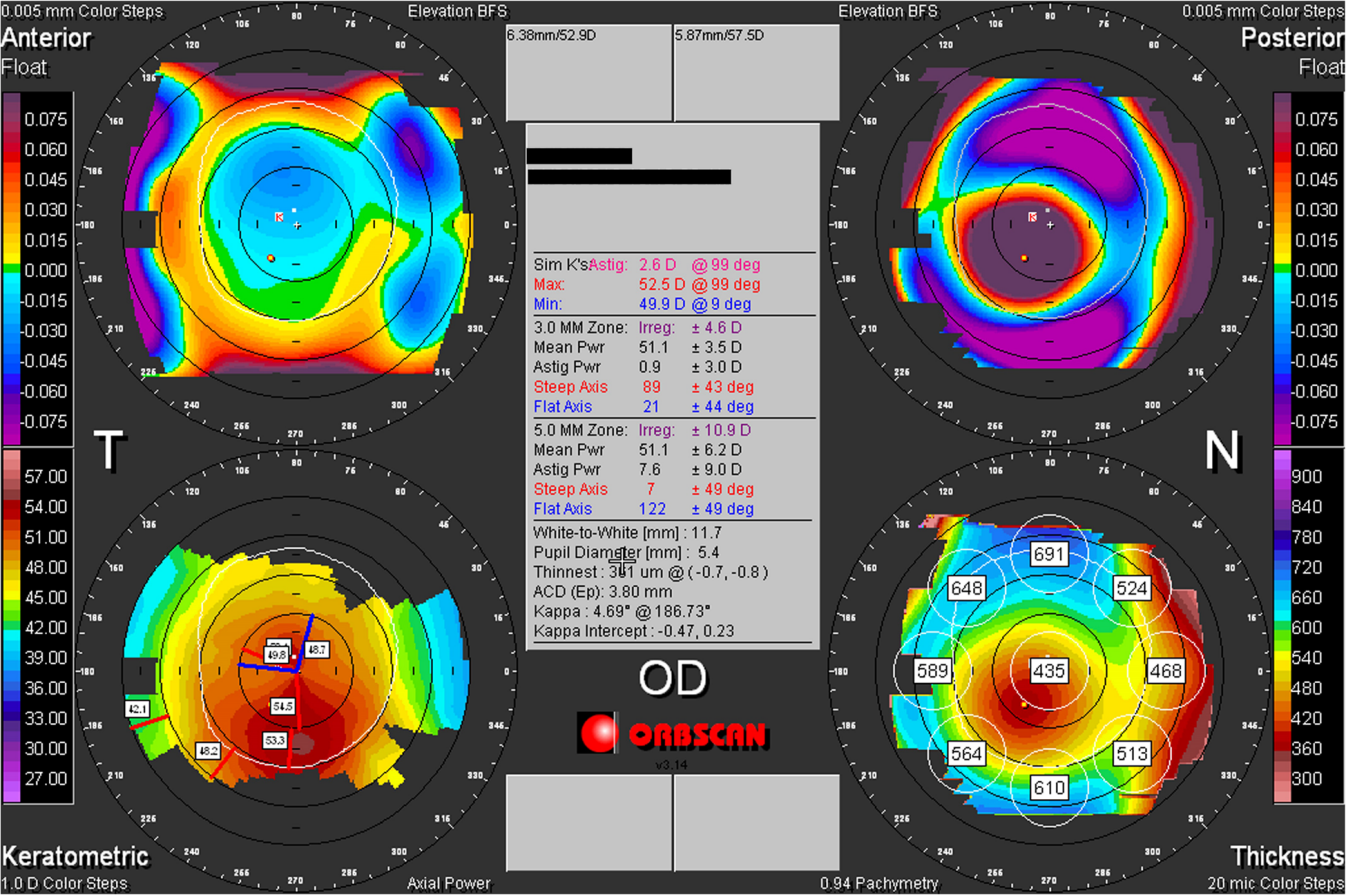
Corneal topographic and pachymetric maps showed significantly steep Sim K values and severe thinning in the inferior paracentral cornea

A 19-year-old male visited the Ophthalmology Department in November 2013 due to a rapid decline in visual acuity within a short period of time. Ocular examination with slit-lamp biomicroscopy showed that the corneas of both eyes were transparent, but the inferior corneal quadrant thinned and bulged slightly, and Vogt’s striae were visible. There was a dense copper-brown depositional ring (KF ring) in the Descemet membrane around the periphery with a mean width of approximately 1.5 mm. The widest point was at the 12 o’clock position, at approximately 2 mm (Fig. 1a). The sclera did not stain yellow, the anterior chamber was clear, the pupils exhibited an equal size and shape and were sensitive to light, the lens was transparent, and there was no obvious abnormality on fundus examination. Corneal topography showed that the Sim K value of the right eye was 52.5D@99/49.9D@9, and the thinnest point of the cornea was 0.8 mm below the center, with a thickness of 361 μm. The Sim K value of the left eye was 55.9D@80/51.2D@170, and the thinnest point of the cornea was 0.7 mm below the center, with a thickness of 336 μm (Fig. 2). The parameters of both corneas were consistent with the typical characteristics of KC.

The patient was referred to the Neurology Department for further consultation because of the existence of KF ring. Serological examination showed that the level of serum ceruloplasmin was low (18 mg/L; normal range: 204–407 mg/L), and the level of urinary copper was high (363 μg/24 h; normal range: less than 150 μg/24 h). Abdominal USG showed fine granular changes in the liver parenchyma. In addition, brain MRI showed abnormal signals indicative of cerebral peduncle and globus pallidus on both sides. However, all physical examinations were negative. Based on these findings, the patient was eventually diagnosed with asymptomatic WD. Over the 5 years since then, the patient has worn RGP lenses, consumed a low-copper diet, and taken penicillamine for copper discharge and zinc gluconate to inhibit copper absorption. Moreover, the removal of copper was occasionally assisted via the administration of dimercaptopropane sulfonate. Recent examinations showed that the color of the KF ring was obviously lightened (Fig. 1b), the corneal morphology and thickness were stable, and symptoms of neurological, mental, and liver damage were negative.

## Discussion and conclusion

In general, KC is a noninflammatory localized corneal thinning and protruding disorder [5], mostly occurring in adolescence. It often causes myopia and irregular astigmatism affecting vision quality. In severe cases, vision can be lost due to the perforation of the cornea. The reported incidence of this disease is 50–230/100,000 individuals [6], without significant differences related to sex. Notably, the incidence in Asian populations is relatively higher [7]. Currently, it is thought that the disease may be related to genetic defects, ocular surface inflammation, and mechanical factors such as long-term frequent blinking and the use of a rigid contact lens [8]. However, the mechanism responsible for its development remains unclear. In most cases, KC is an isolated ocular disease, while a few reports have suggested that KC was associated with other diseases, such as Down syndrome, Leber congenital amaurosis, and certain connective tissue lesions, including mitral valve prolapse [1–3].

WD is a congenital disorder of copper metabolism caused by a mutation in the copper transporter gene ATP 7B, which is autosomal recessive, and it has an incidence rate of approximately 1/30,000–1/100,000 [9]. Copper may be deposited in the liver and brain, causing cirrhosis, acute liver failure, and nonspecific neuropsychiatric symptoms, such as dysarthria, tremors, ataxia, and inability to concentrate. The KF ring is a characteristic manifestation of the disease. It is a brown ring formed by the deposition of copper in the Descemet membrane of the bilateral limbus cornea. The KF ring is observed in most patients with neurological symptoms and in approximately half of those patients without neurological symptoms [10].

Hamid Gharaee [4] reported a 15-year-old female patient with KC combined with KF ring, but her brain MRI, abdominal USG and serological examination did not show typical features of WD until 2 years later. The follow-up treatment and disease progression were not reported in his report. Ours is the second case of WD combined with KC so far. In our case, the MRI, abdominal USG and serological examination were positive when KF ring was first detected, and we followed the patient for over 5 years to observe the changes in WD and KC.

Current research has not suggested any association between the occurrence of KC and WD. KC usually develops rapidly in adolescence and gradually stabilizes after the age of 30. This patient was 19 years old, and he insisted on a low copper diet and received medication to promote copper excretion and inhibit absorption, without any therapy for KC (e.g., corneal collagen cross-linking). Fortunately, his corneal morphology has remained stable for the last 5 years. We surmise that KC remained stable due to active treatment of WD and fading of the KF rings, although there is no evidence that WD with a large amount of copper deposition in the Descemet membrane would change the collagen fiber integrity or biomechanics. We look forward to following up this case with in-depth research results.

## Data Availability

The data and materials are presented within the manuscript.

## Abbreviations

WD: Wilson’s disease
KC: Keratoconus
KF ring: Kayser-Fleischer ring
RGP: Rigid Gas Permeable
